# Study protocol of D1Ce Screen: A pilot project of the Italian national screening program for type 1 diabetes and coeliac disease in the paediatric population

**DOI:** 10.1371/journal.pone.0328624

**Published:** 2025-08-13

**Authors:** Olimpia Vincentini, Flavia Pricci, Marco Silano, Umberto Agrimi, Francesca Iacoponi, Marika Villa, Chiara Porfilio, Adalgisa Ilaria Sedile, Francesca Ulivi, Carlo Catassi, Valentino Cherubini, Antonio D’Avino, Paola Carrera, Vito Lampasona, Emanuele Bosi

**Affiliations:** 1 Department of Food Safety, Nutrition and Veterinary Public Health, Istituto Superiore di Sanità, Rome, Italy; 2 Department of Cardiovascular and endocrine metabolic diseases and aging, Istituto Superiore di Sanità, Rome, Italy; 3 Fondazione Italiana Diabete, Milan, Italy; 4 Department of Paediatrics, Polytechnic University of Marche, Ancona, Italy; 5 Department of Women’s and Children’s Health, Azienda Ospedaliero-Universitaria, Ospedali Riuniti di Ancona, G. Salesi Hospital, Ancona, Italy; 6 Federazione Italiana Medici Pediatri (FIMP), Naples, Italy; 7 Division of Genetics and Cell Biology, Unit of Genomics for Human Disease Diagnosis, IRCCS San Raffaele Scientific Institute, Milan, Italy; 8 Diabetes Research Institute, IRCCS San Raffaele Hospital, Milan, Italy; PLOS: Public Library of Science, UNITED KINGDOM OF GREAT BRITAIN AND NORTHERN IRELAND

## Abstract

**Background:**

The D1Ce Screen pilot study stems from the Italian Republic Law 130/2023 introducing the screening based on autoantibody measurement on capillary blood for the identification of people in pre-symptomatic, early phase of type 1 diabetes (T1D) and/or having silent celiac disease (CD) in the general paediatric population to reduce the impact of the two more frequent and severe chronic diseases of childhood.

**Aim:**

Primary aim is to assess, on a smaller scale, the organizational feasibility, acceptability and sustainability by the National Health Service of the screening program to be conducted nationwide in Italy according to the law.

**Methods and analysis:**

This is an observational multicenter study, planning to screen 5,363 children from four Italian regions (Lombardy, Marche, Campania, Sardinia), proportionally distributed according to population of the single Regions, representative of the North, Centre, South and Islands of Italy. Participants are screened by autoantibodies within three classes of age 2–2.9, 6–6.9, and 10–10.9 years, corresponding to reported peaks of seroconversion, in order to maximize the identification of future cases of disease. HLA typing for HLA DQ2 and DQ8 is also performed for CD risk in dry blood spots (DBS). Screening procedures are conducted by primary care paediatricians (PCPs), responsible for: direct contact with families; information about the study; administration of written informed consent, privacy statement and questionnaires; execution of blood drawing by finger prick; capillary blood collection for autoantibody and HLA testing and shipment to the central laboratory. Feasibility, acceptability and sustainability will be estimated by number of participating paediatricians; screened children in the four Regions and within the classes of age; feedback questionnaires; number of fingerpicks to obtain sufficient capillary blood for assays; any adverse events; costs evaluation in relation to assigned budget. Secondary objectives include frequency of T1D and CD specific autoantibodies to assess the prevalence of pre-symptomatic (Stage 1 and Stage 2) TD1 and undiagnosed CD in the three classes of age of general paediatric population.

**Study registration:**

D1Ce Screen is not registered to any International Study Registry, as it is a pilot observational study requested by the Italian law 130/2023.

## Introduction

### Background

Type 1 diabetes (T1D) and celiac disease (CD) are the most relevant autoimmune chronic diseases in paediatric age [1,2].

T1D is due to an autoimmune destruction of the insulin-producing β-cells within pancreatic islets, leading to progressive insulin deficiency and finally ensuing in hyperglycaemia and other severe metabolic perturbations, with need of exogenous insulin administration for survival [3].

Over the last few decades, several study screening general population or families at risk showed that the clinical onset of T1D is preceded by a long pre-symptomatic phase characterized by the appearance of islet specific autoantibodies, including antibodies to GAD (GADA), insulin (IAA), IA-2 (IA-2A) and ZnT8 (ZnT8A), indicating that the measurement of islet autoantibodies is the most effective tool for identifying individuals at risk and stage pre-symptomatic T1D, ideal for screening purposes [4]. Islet autoantibodies usually develop sequentially and when two or more of these markers are found in children or adolescents without diabetes, this indicates that they are in the pre-symptomatic phase of T1D, with an almost certainty of progression to overt clinical T1D, at least in children and adolescents [5]. The evidence of beneficial effects associated to the screening for the identification of pre-symptomatic disease has been progressively consolidated, resulting in early diagnosis with milder clinical manifestations, including a significantly reduced incidence of DKA, an acute and potentially life-threatening complication occurring at disease onset, and, at the same time, the possibility to consider interventions aimed at slowing the further progression of the disease [6–8].

CD is a chronic inflammation of the small intestine, recognizing a genetic predisposition and an autoimmune mechanism, precipitated by dietary gluten intake [9]. Treatment is lifelong removal of gluten from the diet. Up to 50% of people with CD are undiagnosed, due to mild or atypical symptoms [1]. The diagnostic and predictive marker of CD is tissue transglutaminase autoantibodies (tTGA). Screening for tTGA, IgA and IgG in those individuals with IgA deficiency, is the only tool that can identify all cases of CD [10]. Islet autoantibodies and TGA can be measured as a combined test, also applicable using capillary blood samples.

Although T1D and CD are distinct entities, they can coexist in the same person, accounting for up to 8% of all cases [11]. There are also many similarities: both are chronic autoimmune diseases, associated with the presence of circulating autoantibodies preceding by months or years the clinical manifestations of the disease. Moreover, they partly share a common genetic susceptibility, mainly associated with polymorphisms of the HLA complex [12]. Furthermore, the incidence and prevalence of both diseases have constantly increased over the last decades [13,14], representing a growing burden for public health worldwide.

The evidence suggests, on one hand, that T1D and CD may share common etiological mechanisms; on the other, it underscores the urgent need to develop programs aimed at curbing the spread of these conditions, minimizing their impact on public health and promoting interventions that can change the course of the disease. A possible strategy is the implementation of extensive screening programs in the general population to identify people at increased risk.

### Prior research

Several large screening programs based on autoantibody screening have been implemented over the last two decades [15] including DAISY [16], BABY-DIAB [17], DIPP [18,19], TrialNet [20,21], TEDDY [22], Fr1da [23] and INNODIA [24] for T1D and ASK [25] and TRIAD [26] for combined T1D and CD. Additionally, the possibility of using blood capillary for autoantibody measurement of both T1D [27–29] and CD [30] allowed the widespread adoption of blood capillary sampling for large scale screening of the general population [31]. Furthermore, a clinical trial based on the anti-CD3 monoclonal antibody Teplizumab, demonstrated for the first time in pre-symptomatic T1D the ability to delay the clinical onset of the disease [32,33]. That study was the first concrete example that T1D could one day be prevented.

### Rationale

This rapidly changing scenario is fully acknowledged by the recent Italian Law making screening for T1D and CD available for all children and adolescents nationwide and established as a public health programme [16]. We here report the protocol of the pilot D1Ce Screen (Diabete tipo 1 e Celiachia Screening) study promoted by the Italian Ministry of Health and implemented through its operating arm, Istituto Superiore di Sanità (ISS), investigating feasibility, acceptability and sustainability within the National Health System of the screening program contained in the law. The rationale is to identify the challenges of translating the law content into a Public Health Program.

## Aims

Overall objective of D1Ce Screen is to perform a screening using capillary blood sampling for autoantibody assays and HLA typing to identify children at risk, either in their pre-symptomatic stage of T1D or as silent CD, in the general pediatric population of Italy.

The primary aim is to assess on a smaller scale the organizational feasibility, acceptability and sustainability within the National Health Service of the pediatric screening program contained in the law.

Secondary aims are to estimate the prevalence T1D and CD specific autoantibodies, the frequency of HLA Class II DQ2 and DQ8 haplotypes conferring risk for CD and the association of CD and T1D antibodies with HLA types.

## Materials and methods

Study design

D1Ce Screen is an observational multicenter study conducted in 4 population samples from 4 Regions: Lombardy, Marche, Campania and Sardinia, representative of North, Centre, South and Islands of Italy, respectively.

The screening is conducted using capillary blood samples for the measurement of T1D and CD specific autoantibodies and genetic typing for CD predisposition.

Primary care pediatricians (PCPs) are responsible for the screening procedure, including contact with families and children, execution of capillary blood sampling and its shipment to central lab and associated documental accomplishments. The whole procedure takes place in the paediatricians’ office.

Target of the population screening are children of three classes of age, 2–2.9 year, 6–6.9 years and 10–10.9 years, chosen since they correspond to the peaks of seroconversion to T1D specific autoantibodies [34,35], intercepting also most of the specific autoantibodies developed in the meanwhile [36].

For eligibility, the inclusion criterion is age. Parents, or legal tutors, must have the ability to understand the purpose of the project and sign the written informed consent. The only exclusion criterion is a diagnosis of clinical T1D. Participation of family paediatricians is voluntary, but compensated, while participation by families is only voluntary.

Autoantibody measurements and HLA typing on capillary blood samples are centralized in a single laboratory.

**Fig 1:**
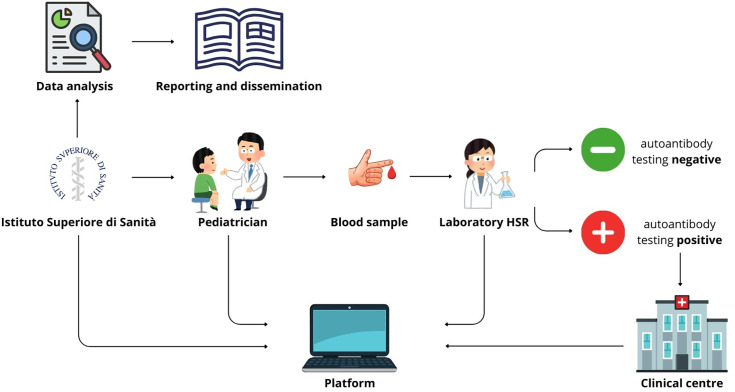
D1Ce Screen workflow.

The D1Ce Screen workflow is described in Fig 1.

## Study setting

### Participant sample definition

**Fig 2:**
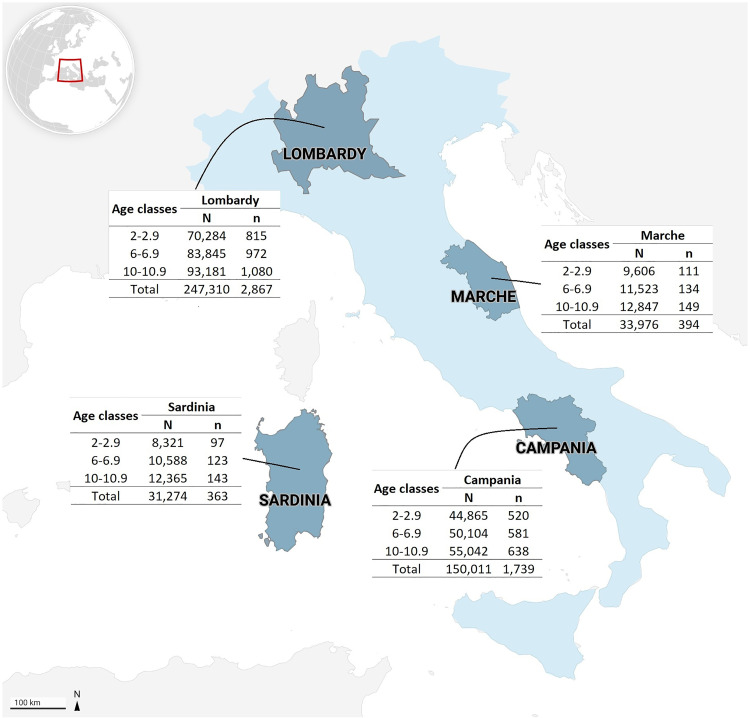
Paediatric sample distribution for each region and age classes (years) involved.

This study is conducted in children residing in the regions of Lombardy, Marche, Campania and Sardinia as of January 1, 2023 [37]. A convenience sample of 5,363 children (1.6% of the Italian paediatric population) will be screened, proportionally distributed and stratified according to the population residing in each region (Fig 2).

### Inclusion and exclusion criteria

The main inclusion criterion is age: children who belong to the three age groups of 2–2.9, 6–6.9 and 10–10.9 years are included. The only exclusion criteria is the diagnosis of T1D. There is no matched control cohort according to study plan.

### Primary care pediatricians

PCPs were preliminary identified through a survey for voluntary participation within their professional associations.

PCPs randomly select participating children within their clinical practice, based on pre-selected classes of age and knowledge of families and their possible positive response to a screening proposal.

PCPs are responsible for direct contact with families, information about the study, administration of written informed consent and questionnaires to families, fulfilment of the ISS central platform and the execution, collection and subsequent shipping of the capillary blood samples obtained by finger-pricks.

PCP will be also responsible for follow up and monitoring of children testing positive at the screening, belonging to families unwilling or unable to engage with specialist care for further monitoring and follow up (see below).

PCP undergoes online training sessions by ISS on scientific and practical aspects of the study, with focus on the ISS-D1Ce website, instruction on D1Ce Screen study platform, access to informative materials for children and families, including flyer and brochure with the Muppets cartoons.

### Information for participant and families

The overview of D1Ce Screen project has been disseminated by the website https://www.iss.it/en/-/il-progetto-d1ce to provide improved levels of awareness and engagement about the relevance of the screening procedure for T1D and CD. In detail, A section of the website is dedicated to children and their families providing a brochure designed by a cartoonist, adopting muppets mimicking the importance and promoting participation to the screening for T1D and CD. A specific section is dedicated to the D1Ce Screen project to update about recruitment and results.

### Capillary blood sampling and shipping

Tailored individual kits for capillary sampling are prepared, containing single-use lancing device (Accu-Check® Safe-T-Pro Plus, Roche, Germany), capillary blood collection microtube 200uL with serum-gel separator (Microvette® 200 Serum Gel CAT, Sarstedt, Germany), Guthrie/Dried Blood Spot (DBS) cards (Spot Saver RUO Card®, Revivity, USA) in specimen transport bag, each marked with a code for the anonymization of the participant enrolled. The paediatrician collects capillary blood samples by fingerpick in the microtube (minimum volume for autoantibody assays 25 µL) and few drops onto DBS (minimum 5 drops for genetic typing) and stores samples in a refrigerator at 3–5°C in special packages and containers provided for biological material. The samples are sent to the laboratory via a specialized courier, with observation of all safety rules preventing biological material dispersion and protecting the environment and transportation and laboratory workers from any risk of contamination.

### Laboratory assays

Autoantibody assays on capillary blood and HLA-DQ typing on DBS are centralized in a single laboratory (Laboratory of Autoimmunity, Diabetes Research Institute and Laboratory of Clinical Genomics, IRCCS San Raffaele Hospital, Milan). Methods for islet autoantibody measurement will be ELISA for combined GADA, IA2A and ZnT8A [38,39] followed, in positive samples, by ELISA for the three single markers. LIPS was also used in parallel, for the three single autoantibodies [40–42] and IAA [43] for confirmation and comparison. TGA-IgA and TGA-IgG will be measured by ELISA, followed by LIPS in positive samples for confirmation. HLA-DQ2 and DQ8 typing will be performed on DBS by Real Time PCR using TaqMan® probes technology for detecting the HLA-DQB1*02, DQB1*03:02 and DQA1*05 alleles (Genvinset® HLA Celiac). The results of the assays are reported into the platform and PCP informs the parents about the tests.

### EDENT1FI registration

All participants stating specific written consent, incorporated within the privacy policy statement, will be offered to be registered with a pseudoanonymised technique in the European Action for the Diagnosis of Early Non-clinical Type 1 Diabetes for Disease Interception (EDENT1FI), a large European project aiming at harmonization and standardization of screening for early-stage T1D throughout Countries [44].

### Feasibility and acceptability questionnaires

Feasibility and acceptability of the screening program for T1D and CD are assessed through questionnaires to PCPs and families, available as online tools.

A questionnaire on evaluation of feasibility and acceptability will be administered to PCPs at the end of study recruitment. The target of PCPs completing the evaluation questionnaires is 75%.

Acceptability for families will be assessed measuring anxiety, depression and quality of life in parents associated with participation in screening of their child. Three existing validated questionnaires will be offered to families: HADS (Hospital Anxiety and Depression Scale) [45], SAI-6 (State Anxiety Inventory 6 items) [46,47] and EQ-5D-5L (EuroQol-5 Dimensions-5 Levels) [48]. Questionnaires will be self-administered after proposal, but with no participation and influence, by PCPs at study entry and following the provision of test results. The target of families undergoing questionnaires evaluation is 2%.

### Regional specialized centers

Participants testing positive for either T1D or CD autoantibodies are referred to regional pediatric specialized centers, identified based on clinical expertise and scientific reputation. They act as referral centers for late follow-up and monitoring of cases identified as at risk. A specific protocol for pre-symptomatic T1D has been developed and published [49]. Participants positive for CD specific autoantibodies will complete clinical assessment at the CD regional specialized centre for possible diagnosis according to current diagnostic criteria [50]. The regional specialized centers also act as support to the PCPs for immediate assistance at the time of autoantibody positivity finding and follow up and monitoring of children from families unwilling or unable to engage with specialist care.

### The D1Ce screen platform, data collection and management

A web-based platform specifically designed for D1Ce Screen has been developed by ISS. Key functions and features, in a user-friendly environment, include: participants data entry and anonymization, personalized dashboards, reporting and documentation in compliance with General Data Protection Regulation. All the collected data will be entered through the D1Ce Screen platform and stored in the database managed by the ISS. The data processing is scheduled at central level (ISS) where data will be also processed and statistically analyzed.

### Study schedule

**Fig 3:**
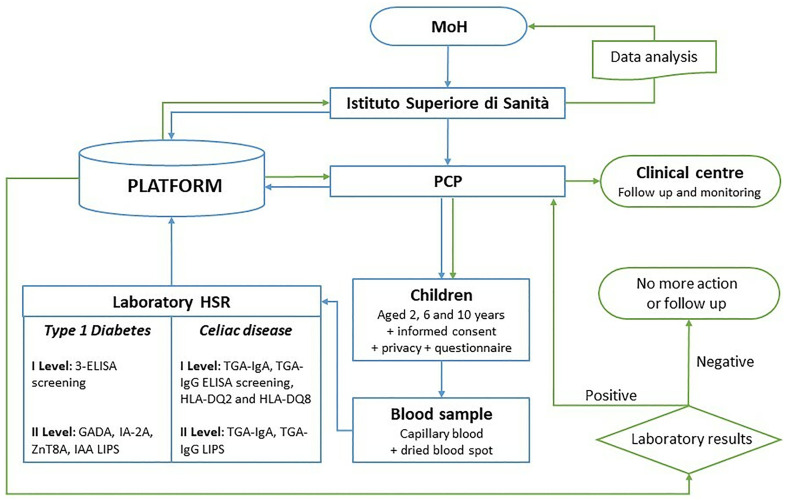
Study flow chart: arrows underline the direction of information flow (pre and post laboratory outcome are blue and green arrows, respectively). HSR: “San Raffaele” Hospital-IRCCS; PCP: primary care paediatricians; MoH: Italian Ministry of Health.

The study lasts a total duration of 20 months. The pre-screening phase has been completed: first two months of the study have been dedicated to the identification of volunteer PCPs, identification of the regional specialized centers, design of acceptability questionnaires for PCPs and families, setting up and online publication of the web site https://www.iss.it/d1ce-screen-copertina, preparation of information material for children and families, design of the Web-Based Platform, preparation of educational material for the volunteer PCPs, online training sessions for each region by ISS experts to participating PCPs, setting up and distribution of the tailored kit for capillary blood sampling. The screening campaign started in April 2024 with the first recruitment on May 14^th^ 2024 and will end on March 31^th^ 2025 (Table 1). During the enrollments, data are collected in the platform, followed by an additional period of 8 months for data analysis and referral of participants identified as early-stage T1D and undiagnosed CD to regional specialized centers. The study flow chart is reported in Fig 3.

**Table 1:** Regional recruitment and specialized centres.

REGION	FIRST RECRUITMENT	END OF RECRUITMENT	TD1-SPECIALIZED REGIONAL CENTER	CD-SPECIALIZED REGIONAL CENTER
Lombardy	16/05/2024	31/03/2025	Diabetes Research Institute, IRCCS San Raffaele Hospital, Milan	-Paediatric Unit IRCCS “San Raffaele” Hospital, Milan
Marche	17/05/2024	31/03/2025	Department of Women’s and Children’s Health, G. Salesi Hospital, Ancona	Department of Paediatrics, Marche Polytechnic University, Ancona
Campania	14/05/2024	31/03/2025	Regional paediatric diabetes centers: Department of Paediatrics, “Luigi Vanvitelli” Campania University, Naples Department of Translational Medical Science, Paediatric Unit, Federico II, University Naples	Regional celiac disease center: Paediatric Unit “Federico II” University Hospital, Naples
Sardinia	24/05/2024	31/03/2025	Paediatric unit-Diabetology, “A. Cao” Microcitemico Hospital, Cagliari	Paediatric Unit-Gastroenterology, “A.Cao” Microcitemico Hospital, Cagliari -

## Outcome measures

### Primary

Primary outcome measures include number of participating PCPs; number of screened children in the four Regions and within the three classes of age; feedback questionnaires from family pediatricians and children’s parents to assess the feasibility and acceptance of screening both by physicians and families; number of fingerpicks in each case to obtain sufficient capillary blood volume for assays and possible need for venous sampling; proportion of successful sample collection with sufficient blood volume for autoantibody measurement and genetic typing; adverse events associated with screening-procedures; costs evaluation.

### Secondary

Secondary outcome measures include prevalence of T1D associated antibodies (GADA, IA-2A, IAA and ZnT8A), occurring as single or multiple autoantibodies, and of CD associated antibodies (TGA); frequency of HLA Class II DQ2 (DQA*0501-DQB*0201) and DQ8 (DQA*0301-DQB1*0302) haplotypes conferring risk for CD; association between CD and T1D antibodies and HLA types; identification of pre-symptomatic T1D suitable to educational programs, monitoring and follow up for DKA prevention and access to disease modifying therapies; prompt treatment of newly diagnosed CD by gluten free diet; prevention of non-gastrointestinal complications of CD.

### Data management plan

All data are collected, registered and managed in the web-based D1Ce Screen platform. These include: participants and families baseline characteristic and signed written informed consent collected by the PCPs; self-administered family questionnaires on acceptability; feasibility and acceptability questionnaires administered to PCPs; results of T1D and CD specific autoantibody measurement and HLA type from the centralized laboratory; referral of baseline characteristics and autoantibody and HLA information to the expert regional centers for subsequent monitoring and follow up [49]. Once data analysis is completed, findings will be reported to the competent policy makers and for publication in international scientific journals and/or they will be shared anonymously during professional activities. After the end of the study, full protocol and data will be available on request to the principal investigator. Copies of informed consent for participants will be also provided.

### Potential bias

A possible selection bias could be a greater willingness to participate on the part of families at risk (parents with diabetes or celiac disease). A dedicated question is included in the recruitment questionnaire.

Capillary blood collection may be subject to differences in technique and sample quality by paediatricians (sampling bias) and logistical issues (transport, temperature, etc.), which could affect volume adequacy and quality of the results. Standardized training will be provided to all paediatricians involved in the study to ensure consistent sample collection procedures. Finally, the dedicated platform ensures uniform data collection and entry across all participating centres but does not prevent data entry errors by introducing a reporting bias.

### Safety considerations

No substantial study-related adverse events (AEs) are expected, with the exception of some pain and bruising at the site of blood sampling and possible vagal reactions in participants on the occasion of the blood capillary drawing. Nonetheless, a system for the collection, management, and reporting of AEs and serious SAEs was implemented to ensure participant safety and regulatory compliance.

### Statistical analysis plan

Data will be reported as mean and standard deviation or as median and interquartile range, depending on the distribution of continuous variables. Discrete and categorical variables will be reported as absolute frequencies and percentages. Descriptive analyses will be performed stratifying by region of residing, sex and age. Depending on observed prevalence of T1D and CD, possible risk factors (sex, familiarity etc.) will be investigated using correlations and associations with presence/absence of autoantibodies. The Pearson correlation coefficient and Chi square test will be applied to examine correlations and associations, respectively. Also, will be evaluated simultaneous presence of T1D and CD. A p value <0.05 will be considered statistically significant and all analyses will be conducted on statistical software R v.4.4.0 (https://www.r-project.org/) and Microsoft Excel (Microsoft Office 2016).

### Involvement of patient/participant representatives in protocol development

Although for the D1Ce Study no Patient Advisory Board (PAC) has been appointed, a representative of Fondazione Italiana Diabete (FID) collaborated to all phases of the design of the study, providing consultancy for informative materials and communication to families, including questionnaires to families to ensure comprehensibility for lay people and independence from PCPs influence during the completion procedure.

### Ethical considerations

The D1Ce Screen study and Privacy policy, formerly evaluated and authorized by the Data Protection Office, were approved by the National Ethics Committee for Public Bodies, Rome, 22 December 2023 (Protocol PRE BIO CE No. 0059835). Participants will be clearly and widely informed regarding all aspects of the study. Both parents and legal guardian of participants will sign the written informed consent and privacy statement. The collection and storage of personal health data will be limited to study-related information and may not be disclosed, made available or otherwise used. All laboratory samples, reports, data collection and administrative forms will be identified by a coded ID number to maintain participant confidentiality. Only authorized personnel can link this code to the participant identity. All the information will be stored in the D1Ce platform with limited access and will be protected by a password and username access system. Study participant information will not be disclosed outside the study without the written authorization of the participant, including for follow up by reference regional centers. The results of the study will be communicated to an ad hoc Observatory required by the law and nominated by the Ministry of Health.

## Discussion

### Strengths and limitations

The D1Ce Screen study is a pilot observational study promptly implemented by the Italian Ministry of Health in response to the approval of the law on the general screening of T1D and CD in pediatric ages, to test feasibility, acceptability and sustainability of the screening program contained in the law. Strengths of the protocol include: the combined screening for T1D and CD in the general pediatric population from four sample regions in Italy; the evaluation of feasibility acceptability and sustainability of a unique nationwide screening program introduced by the law; the screening execution by a network of PCPs using blood capillary samples, analyzed centrally by a single laboratory. Limitations include the relatively limited number of screened children for a general population study and the screening around only three classes of age across the entire pediatric age range. Critical feedbacks on feasibility and acceptability are expected by the D1Ce Screen, valuable to finalize operability and functionality of the National Screening Program starting in early 2025.

### Dissemination plan

The results of the study will be communicated to the competent policy makers, specifically to the Ministry of Health and to the ad hoc Observatory, as requested by law. Results will be published in scientific journals and presented at scientific conferences and meetings for lay people/population/schools in order to increase awareness around T1D and CD in Italy.

## Supporting information

S1File SPIROS check list. (DOCX)
