# Supplementary material for: Study protocol of D1Ce Screen: A pilot project of the Italian national screening program for type 1 diabetes and coeliac disease in the paediatric population
Source: PLoS One. 2025 Aug 13;20(8):e0328624. doi: 10.1371/journal.pone.0328624 (PMC12349695; doi:10.1371/journal.pone.0328624)
Supplement: S1File — (DOCX) [file pone.0328624.s001.docx]

Standard Protocol Items for Observational Studies (SPIROS)

**Table** **1**: Checklist of preliminary items

| **Section and topic** | **Description / sub-categories** | **Addressed on page number** |
| --- | --- | --- |
| **i)** **General** **Information** | | |
| Title | Descriptive title identifying study design | Page 1 of 14; Lines: 1-2 |
| Protocol version | Version or amendment number and date and summary of changes | NA |
| Protocol summary | Brief summary of protocol research | Page 2 of 14; Lines: 37-62 |
| Sponsor and partner  institute name | Name of sponsor and participating institutes (if applicable) | Page 1 of 14; Lines: 28-29 |
| Investigators name | Name of principal and co investigators. | Page 1 of 14; Lines: 3-5 |
| Affiliation of  investigators | Affiliated institutions of investigators | Page 1 of 14; Lines: 8-17 |
| Principal researcher  contact detail | Name, email address, affiliation of Principal researcher for correspondence. | Page 1 of 14; Lines: 22-26 |
| Table of content | Table of content | NA |
| Page number | Page number on each page of protocol | Pages 1-14 |
| List of Abbreviations | A detailed List of all abbreviations used in protocol with full form. | NA |
| **ii)** **Introduction** | | |
| Background of study | Scientific background of study | Page 2-3 of 14; Lines: 69-102 |
| Review of prior  research | Summary of all previous relevant research | Pages 3 of 14; Lines: 104-112 |

| Rationale of study | Justification for conducting the study | Page 4 of 14; Lines: 114-120 |
| --- | --- | --- |
| Aim | Broader aims and specific objectives of the study | Page 4 of 14; Lines: 127-130 |
| Objective of study | Primary and secondary objectives of study | Page 4 of 14; Lines: 124-126 |
| Prespecified  hypothesis | Prespecified null or alternative hypothesis | NA |
| **iii)** **Methods** | | |
| Study design | Description of type/design of study | Pages 4 of 14; Lines: 132-148 |
| Study setting | Description of setting, locations, relevant dates, including periods of recruitment/survey, exposure, follow-up, and data collection.  Schedule of study procedure – Figure or table | Pages 5-7 of 14; Lines: 153-240  Page 4 of 14; Figure 1; Lines 150  Page 5 of 14; Figure 2; Lines 159 |
| Sample size | Estimated number, calculation and assumptions  Power calculation | Page 5 of 14; Lines: 156 |
| Sampling procedure | Description of sampling strategy to ensure representativeness and control  of potential bias | Page 5 of 14; Lines: 156-157 |
| Participants | Cohort study—eligibility criteria, and the sources and methods of selection of participants. Describe methods of follow-up.  For matched studies, give matching criteria and number of exposed and unexposed  Case-control study—Give the eligibility criteria, and the sources and methods of case ascertainment and control selection. Give the rationale for the choice of cases and controls  For matched studies, give matching criteria and the number of controls per  case | Page 5 of 14; Lines:161-164  NA  NA |

|  | Cross-sectional study—Give the eligibility criteria, and the sources and methods of selection of participants | NA |
| --- | --- | --- |
| Variables | - All outcomes - Exposures- definition of exposure of interest, - Predictors - Potential confounders - Effect modifiers | Page 8 of 14; Lines: 266-273  NA  NA  NA  NA |
| Data Sources/ Measurement | - For each variable of interest, give sources of data and details of methods of assessment (measurement). - Describe comparability of assessment methods if there is more than one group - Data collection points table - Blinding procedure | Page 8 of 14; Lines: 270-276  NA  NA  NA |
| Bias | Describe any efforts to address potential sources of bias  More specifically-   - Information bias - Selection Bias - Control for confounding | Page 9 of 14; Lines: 291-298  NA  NA  NA |
| Statistical analysis plan | - Method of primary / secondary outcomes and additional analysis - Handling of missing data - Post-hoc analysis | Page 8-9 of 14; Lines: 298-307  NA  NA |

| Handling of withdrawals and lost to follow up | Describe the procedures to be followed when a participant ceases participation in the study prematurely or is lost to follow up | NA |
| --- | --- | --- |
| Replacements | Provide information on whether or not participants who discontinue the study will be replaced via additional recruitment to maintain the required sample size. | NA |
| Outcome | Define and describe all primary and secondary outcome or lost to follow-up | Page 8 of 14; Lines: 266-273 |
| Database management | Detail plan of database management including:   - Data collection (electronic or paper based) - Source data - Data entry - Data editing - Coding - Data storage - Record retention - Data confidentiality | Page 7 of 14; Lines: 240-246  Page 9 of 14; Lines: 281-290 |
| Validation of instrument | Reliability / validity of instrument or plan to establish validation | NA |
| Follow up | Plan of follow up and addressing lost to follow up | Page 7 of 14; Lines: 231-238 |
| Quality control | - Method of quality control - Monitoring (internal and external) - Training of surveyors | NA  NA  NA |
| Quality assurance | Plan of quality assurance | NA |

| Expected outcome/results | A brief description of expected outcome or results | Page 10 of 14; Lines: 347-349 |
| --- | --- | --- |
| **iv)** **Ethical** **consideration** | | |
| Ethical approval | Weather it has been obtained and name of ethical committees. If approval not sought, Reason | Page 10 of 14; Lines: 324-326 |
| Agreement and consent | Method of taking consent. Reason if consent not sought | Page 10 of 14; Lines: 326-327 |
| Risk / Harm to participants | Any potential risk or harm to study participants | Page 9 of 14; Lines: 300-304 |
| Adverse event and Severe adverse event reporting | Outline how Adverse Event and Severe adverse event information will be collected. | Page 9 of 14; Lines: 300-304 |
| **v)** **Reporting** **and** **dissemination** | | |
| Protocol amendments | Methods of communicating to investigators/IRBs and documenting | NA |
| Dissemination | How results will be disseminated to participants, practitioners, public | Page 10 of 14; Lines: 352-356 |
| Publication Plan | Who has right to publish; restrictions; authorship guidelines  Open Access | Page 1 of 14; Lines: 32-36  Page 10 of 14; Lines: 354-356 |
| Reporting of early stopping | Dissemination of results if trial is stopped early (for any reason) | NA |
| **vi) Others** | | |
| Limitations | Limitations of proposed study, including risk of bias | Page 10 of 14; Lines: 346-349 |
| Strength of study | Highlight strengths of proposed study | Page 10 of 14; Lines: 342-346 |
| References | List of references cited in protocol | Page 11-14 of 14; Lines: 381-529 |

| Data collection forms | Summary table of all forms used for data collection at each point of study | NA |
| --- | --- | --- |
| Informed consent forms | Sample of informed consent form, translated into local language | supplementary file 2 (S2) |
| Funding | Source of funding and the role of the funders for the present study | Page 1 of 14; Lines: 28-29 |
| Acknowledgement for protocol development | Acknowledgement of persons involved in protocol preparation | Page 11 of 14; Lines: 369-372 |
| Data sharing policy | To describe how data will be made available in public domain. | Page 9 of 14; Lines: 287-290 |
| Contributions of authors to protocol | Listed authors should have participated sufficiently in preparation of  protocol with details of their contribution. | Page 11 of 14; Lines: 358-367 |
| Trial registry | For observational studies also registered as trial | Page 2 of 14; Lines: 64-66 |
| Annexures | Data collection form /instruments Informed consent form  Standard operating procedures (SOPs)  Detailed Statistical analysis plan (SAP) | supplementary file 3 (S3)  NA  NA |
